# Metagenomic Characterisation of the Viral Community of Lough Neagh, the Largest Freshwater Lake in Ireland

**DOI:** 10.1371/journal.pone.0150361

**Published:** 2016-02-29

**Authors:** Timofey Skvortsov, Colin de Leeuwe, John P. Quinn, John W. McGrath, Christopher C. R. Allen, Yvonne McElarney, Catherine Watson, Ksenia Arkhipova, Rob Lavigne, Leonid A. Kulakov

**Affiliations:** 1 School of Biological Sciences, The Queen’s University of Belfast, Belfast, Northern Ireland, United Kingdom; 2 Agri-Food & Biosciences Institute, Belfast, Northern Ireland, United Kingdom; 3 Laboratory of Gene Technology, KU Leuven, Leuven, Belgium; Universiteit Utrecht, NETHERLANDS

## Abstract

Lough Neagh is the largest and the most economically important lake in Ireland. It is also one of the most nutrient rich amongst the world’s major lakes. In this study, 16S rRNA analysis of total metagenomic DNA from the water column of Lough Neagh has revealed a high proportion of Cyanobacteria and low levels of Actinobacteria, Acidobacteria, Chloroflexi, and Firmicutes. The planktonic virome of Lough Neagh has been sequenced and 2,298,791 2×300 bp Illumina reads analysed. Comparison with previously characterised lakes demonstrates that the Lough Neagh viral community has the highest level of sequence diversity. Only about 15% of reads had homologs in the RefSeq database and tailed bacteriophages (*Caudovirales*) were identified as a major grouping. Within the *Caudovirales*, the *Podoviridae* and *Siphoviridae* were the two most dominant families (34.3% and 32.8% of the reads with sequence homology to the RefSeq database), while ssDNA bacteriophages constituted less than 1% of the virome. Putative cyanophages were found to be abundant. 66,450 viral contigs were assembled with the largest one being 58,805 bp; its existence, and that of another 34,467 bp contig, in the water column was confirmed. Analysis of the contigs confirmed the high abundance of cyanophages in the water column.

## Introduction

Lough Neagh is the largest freshwater lake in the British Isles. It is located in Northern Ireland about 30 km to the west of Belfast (54°37′06″N, 6°23′43″W) and has dimensions of 30 km by 15 km. With a mean depth of just 9 m, and a surface area of 392 km^2^, the relatively high mean wind speeds locally (>4.5 m sec ^-1^) ensure that the 3.5 km^3^ of water it contains is completely mixed; oxygen saturation levels rarely drop below 60%. Lough Neagh serves as a main source of potable water in Northern Ireland, providing more than 40% of the region’s supply. Among its other uses, the lake contains Europe’s largest eel fishery, provides sand for the construction industry and offers many tourism and leisure activities. Full details of the lake and of its catchment can be found in [1].

Lough Neagh also has a long history of cultural eutrophication; it receives discharges from several wastewater and sewage treatment plants and from diffuse agricultural sources across its catchment of 4,500 km^2^ with a population of 390,000 [2]. This has caused a shift from mesotrophic conditions at the start of the 20^th^ century to its present status as one of the world’s most hypertrophic lakes–a situation that threatens to irreversibly change its ecosystem. For example, algal species richness has decreased over the last century, with a progressive increase in the dominance of cyanobacteria, most recently of non-diazotrophic species [2]. Although the ecology of Lough Neagh has been studied extensively during the last several decades, little is known about its total bacterial populations [3], whilst the viral community of Lough Neagh has never been studied, even though this is likely to make a major contribution to nutrient cycling in the lake.

Bacteriophages represent the most numerous and important constituents of microbial communities and are likely to play an extremely important role in the cycling of nutrients [4, 5]. As a result, metagenomic analyses supported by next generation sequencing have been widely conducted in marine environments, but freshwater viromes have so far attracted much less attention. Among the first studies in this area was an investigation of viral communities in fish ponds [6], followed by the characterisation of RNA viromes from a freshwater lake [7] and the profiling of viral diversity in Lake Limnopolar (Byers Peninsula, Antarctica) [8]. Viral metagenomic studies have also been carried out on four freshwater ponds located in the Sahara Desert [9], on Feitsui freshwater reservoir in North Taiwan [10], and at two sites in the aquaculture facility of Kent SeaTech Corporation in California, USA [11]. The detailed study reported in [12] demonstrated the relatedness of viromes from two temperate but ecologically different French lakes, and their genetic distinctiveness from other aquatic communities. Among their findings was the demonstration of similarities in viromes from related environments (freshwater, marine, hypersaline), with the salinity level of the habitat having more impact on the viral community structure than its geographical location. Only one preliminary investigation of the composition of planktonic viral communities in a eutrophic freshwater environment has been carried out to date [13].

In the present study, we report a comprehensive characterisation of the viral and bacterial metagenomes of the water column of Lough Neagh, using Illumina high-throughput shotgun sequencing and 16S rRNA gene targeted 454 pyrosequencing, respectively. We present the identification of the major taxonomic groups and functional categories of the viral community, an analysis of sequences of bacterial origin found in the virome, and a comparison of these to the available datasets from other studies. This study provides a first insight into the structure of the bacterioplankton population and that of its phages in one of the most important European temperate eutrophic freshwater lakes.

## Results and Discussion

### Bacterial diversity

The Lough Neagh ecosystem has been extensively monitored for the last fifty years. The analysis of the monitoring data records, available from literature microbiological and microscopic evidence, and sequencing data allowed us to conclude that major changes in the structure and composition of Lough Neagh bacterial community occur during the spring time (transition to cyanobacterial dominance, see below), which was the reason to use a sample obtained in April for metagenomic analysis of the microbial communities of this lake. The values of chemical and environmental parameters at the time of sample collection (S1 Table) confirmed the typical hypertrophic status of Lough Neagh.

The study of bacterial community structure was based on the pyrosequencing of 16S rRNA gene amplicons and the analysis of the dataset obtained was performed by QIIME [14] as described in the Experimental procedures section. Amplicon sequencing generated 3,275 high-quality reads, of which 2,335 reads were clustered into operational taxonomic units (OTUs) with at least four reads per OTU; a total of 118 different OTUs were identified. 375 reads (16.1%) in 35 OTUs could not be assigned taxonomy by the RDP classifier of QIIME and were designated as “unclassified”. The representative sequences of each OTU were extracted from the dataset and manually examined by carrying out BLASTn [15, 16] searches against the nucleotide collection (nt) database. The inspection of the alignments generated by BLAST for these reads revealed their homology (e-value < 10^−5^) to sequences annotated as 16S rRNA genes of uncultured bacteria and to 18S rRNA gene sequences of various eukaryotic planktonic microorganisms (*e*.*g*., diatoms). The presence of eukaryotic small-subunit ribosomal RNA gene sequences in the amplicon dataset can be explained by non-specific amplification due to the similarity of certain 16S rRNA gene primer sequences to specific regions of 18S rRNA genes, a situation which has previously been observed in metagenomic studies [17, 18]. The unclassified sequences were excluded from further analyses, and the remaining 1960 reads (83.9%), representing 83 OTUs, were assigned taxonomic classifications (to genus level wherever possible).

**Fig 1:**
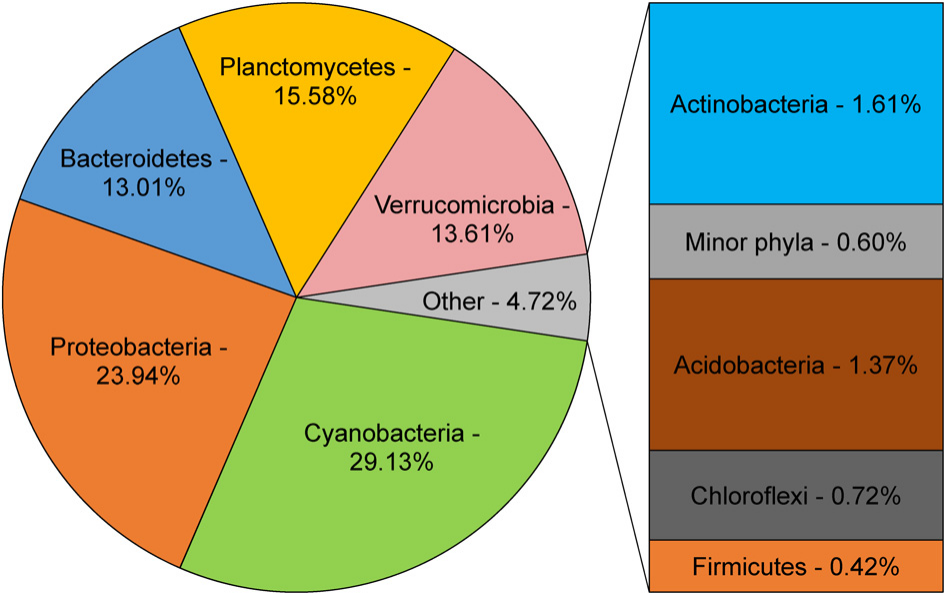
Major bacterial groups found in Lough Neagh (phylum level). Partial sequences of 16S rRNA genes were amplified and sequenced using 454 pyrosequencing. The sequences were clustered into OTUs at the 97% sequence similarity level and taxonomic annotation of OTUs was carried out using QIIME; the results obtained were used to generate the distribution of bacteria at the phylum level.

Bacteria of nine phyla were present in the Lough Neagh water column sample (Fig 1); of these, Cyanobacteria was the most abundant group, comprising 29.1% of the processed reads. While the initial taxonomic classification assigned 39.4% of amplicons to Cyanobacteria, the examination of the taxonomic breakdown of Cyanobacteria at different levels in the QIIME output revealed that about a third of these reads originated from the 16S rRNA genes of Stramenopiles (Heterokonta) and Chlorophyta chloroplasts, which were classified as Cyanobacteria by QIIME algorithms. The chloroplast-related sequences were removed from the subsequent taxonomic analyses of the bacterial community by filtering the OTU table with QIIME scripts and the statistics were updated to reflect that. The remaining 1675 reads were clustered into 74 OTUs, and diversity estimates calculated after rarefaction: Shannon index (H) = 4.679, Simpson index (D) = 0.918. Members of the phylum Proteobacteria accounted for 23.9% of all amplicons, followed by Planctomycetes (15.6%), Verrucomicrobia (13.6%), and Bacteroidetes (13.0%). Other bacterial phyla present in the Lough Neagh water column community (Actinobacteria, Acidobacteria, Chloroflexi, and Firmicutes) constituted less than 5% of the total. Bacterial community structure was clearly dominated by Cyanobacteria, while only 1.7% of 16S rRNA gene amplicons were affiliated with Actinobacteria. At the lowermost (genus) level, two cyanobacterial genera, *Planktothrix* and *Pseudanabaena*, accounted for 18.6% and 8.5% of all sequences, respectively. Verrucomicrobia were mainly represented by genera *Candidatus Xiphinematobacter* (6.1%) and *Luteolibacter* (3.3%). The majority of the reads assigned to Proteobacteria (18.1%) were from 16S rRNA gene amplicons of bacteria of Pelagibacteraceae (SAR11) family [19, 20], the freshwater members of which belong to the LD12 clade [21, 22]. As the RDP classifier was unable to classify the OTU to the genus level, we performed a manual alignment by BLASTn online [23] (http://blast.ncbi.nlm.nih.gov/Blast.cgi, bl2seq megablast algorithm with default parameters) of the representative sequence of the OTU to the prototypical LD12 sequence (Genbank accession no. Z99997.1, data not shown), which demonstrated 99.8% identity of the sequences analysed.

**Fig 2:**
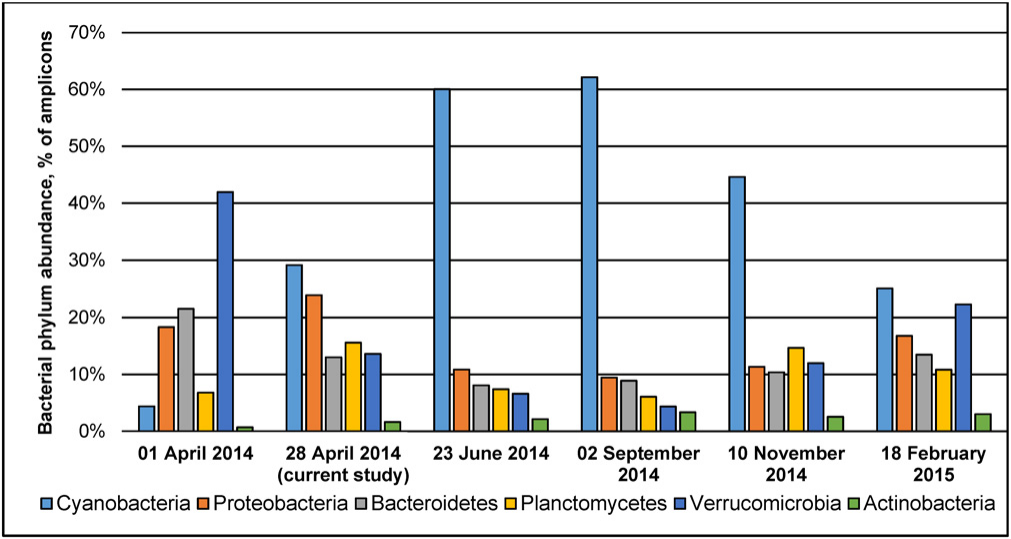
Seasonal changes in abundance of six major bacterial phyla in Lough Neagh over 12-month period. Partial sequences of 16S rRNA genes from the additional water samples collected on 1 April 2014, 23 June 2014, 2 September 2014, 10 November 2014, and 18 February 2015 were amplified and sequenced using 454 pyrosequencing. The sequences were clustered into OTUs at the 97% sequence similarity level and taxonomic annotation of OTUs was carried out using QIIME; the results obtained were used to generate the distribution of bacteria at the phylum level.

To highlight the major changes occurring in the bacterial community of the lake during the year, we performed a similar sequencing analysis of Lough Neagh water samples conducted over 12-month period. The analysis confirmed that indeed Cyanobacteria was the most abundant group in the lake and that the proportion of Actinobacteria remained relatively low (Fig 2).

A comprehensive meta-analysis of freshwater bacterial compositions by Newton and colleagues [24] demonstrated the abundance of Actinobacteria species (over 25% of all 16S rRNA gene sequences on average). Such a predominance of Actinobacteria (over 35%) is characteristic for both of the most comprehensively studied lakes, Bourget and Pavin [25–27]. Notably, only 1.6% of Lough Neagh 16S rRNA gene amplicons showed similarity to this taxon. Using principal coordinates analysis, we performed a comparison of bacterial communities from a range of freshwater environments with publicly available 16S rRNA amplicon datasets and differing in their trophic status and geographic location (S1 File). The analysis conducted demonstrated that Lough Neagh is clearly distinct from the other freshwater lakes analysed. Actinobacteria abundance in bacterial community structures analysed ranged from 1.4% (Lake Michigan) to 76.1% (Ouagadougou reservoir), averaging at 50.5% (95% confidence interval 38.4% to 62.6%). In contrast, the analysis of bacterial composition of Lough Neagh at 6 different time points (Fig 2) demonstrated that Actinobacteria content was in the range from 0.7% to 3.3% throughout the year, being on average just 2.2% (95% confidence interval 1.4% to 3.0%). It is known that Actinobacteria are less abundant in nutrient-rich environments due to their slower growth and decreased competitiveness [28]. It was also recently suggested that the abundance of Actinobacteria negatively correlates with that of Cyanobacteria and the increase of cyanobacterial numbers may reflect serious ecological damage to freshwater systems [29]. High abundance of organic matter, readily available inorganic nutrients (especially N and P), and increased temperatures lead to uncontrollable growth of Cyanobacteria. Cyanobacterial blooms have a number of detrimental effects on an aquatic ecosystem, the most prominent of them being an increase in water turbidity, release of cyanobacterial toxins, and oxygen depletion [30, 31]. All these factors negatively affect the biodiversity of the ecosystem, threatening to cause an irreversible alteration in community structure and composition. Therefore, the dominance of cyanobacteria is an important indicator of deteriorating ecological situation in freshwater environments. Indeed, Cyanobacteria was the largest taxon in terms of 16S rRNA gene amplicon numbers found in the Lough Neagh metagenome in the present study (29.1%; Fig 1) and remained the dominant group of bacteria in all samples studied, except that of 1 April 2014 (Fig 2). A strong correlation between the levels of nitrogen pollution and predominance of Cyanobacteria (more specifically, *Planktothrix*) in Lough Neagh was previously demonstrated [2]. Our analysis of bacterial populations in six timepoints (2014–2015) corroborates the above conclusion.

### Viral community

#### MetaVir analysis of unassembled reads

2,295,055 reads were uploaded to the MetaVir server [32, 33] for taxonomic annotation and comparative analyses with other viromes. Rarefaction analysis was performed on the whole dataset with clustering of sequences at 90% identity level, and demonstrated that, while sequencing effort was substantial and sufficient for accurate taxonomic annotation of major groups of viruses, it wasn’t exhaustive, as the rarefaction curve had not approached a plateau (S1A Fig). To further assess cluster richness, we conducted a comparative rarefaction analysis of subsamples from the Lough Neagh virome and several viral freshwater metagenomes. Comparison with the freshwater lakes Bourget and Pavin is shown in S1B Fig (sampling depth– 50,000 reads, clustering of sequences at 90% identity level). All three rarefaction curves could be fit to linear functions using GraphPad Prism (r^2^ > 0.99); the comparison of their slopes demonstrated that all three curves were different (p < 0.0001) with Lough Neagh having a more diverse virome.

**Fig 3:**
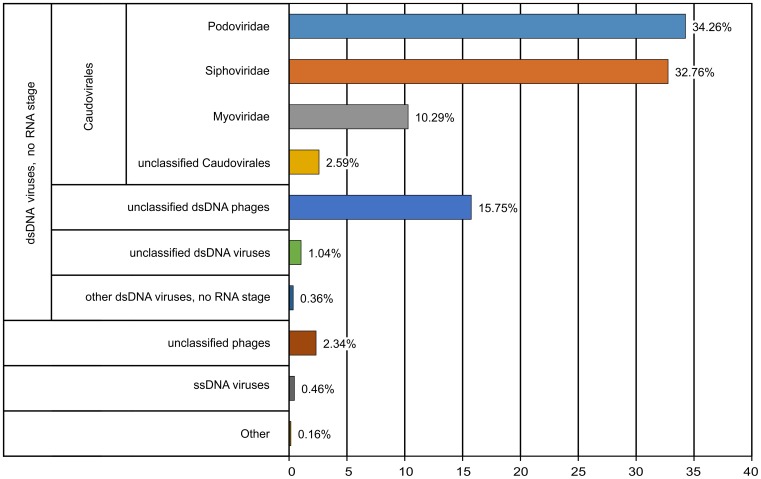
Taxonomic composition of Lough Neagh virome. Composition was computed at the MetaVir server from a BLAST comparison with the RefSeq complete viral genomes protein sequences database. Abundance of the major viral groups shown with the numbers of mapped sequences at the right ends of the corresponding bars.

Taxonomic annotation on Metavir was performed by comparing all reads from the Lough Neagh virome with the RefSeq complete viral genomes protein sequence database (2014-09-10 release) using BLASTx [16]. 14.6% (334,507 reads) of the virome sequences produced a database hit (threshold of 50 on the BLAST bit score, with no minimum alignment length). These reads were annotated on the basis of their similarity to known viruses, and the taxonomic composition of the virome was determined after normalisation with the Genome relative Abundance and Average Size (GAAS) tool [34] to account for differences in the genome lengths of viruses (Fig 3). Less than 0.5% of these reads had similarity to ssDNA viruses, and the majority of the remaining reads (97.0%) originated from dsDNA viruses, of which *Caudovirales* (tailed bacteriophages) accounted for 79.9% of reads. Unclassified dsDNA phage sequences comprised 15.8%, and unclassified dsDNA viruses 1.0% of reads. The majority of reads annotated as arising from *Caudovirales* had similarity to genomes of the *Podoviridae* family phages (34.3% of all reads), closely followed by *Siphoviridae* (32.8%), while *Myoviridae* was the least numerous group, with 10.3% of reads affiliated with this taxon. The predominant subfamilies/genera (accounting for more than 0.5% of metagenome) for *Podoviridae* were unclassified and unassigned *Podoviridae* (26.6% and 0.8%, respectively), *Bppunalikevirus* (2.3%), *Autographivirinae* (1.8%), *P22likevirus* (0.8%), *Epsilon15likevirus* (0.7%), and *Luz24likevirus* (0.5%). The majority of the reads assigned to *Siphoviridae* were from unclassified *Siphoviridae* (29.1%), followed by *Lambdalikevirus* (1.8%), *Phic3unalikevirus* (0.9%), and *Yualikevirus* (0.8%). In the case of *Myoviridae*, no subgroup with abundance of more than 0.5% (except unclassified *Myoviridae*; 8.4%) was identified. Fourteen individual phage sequences were most abundant in the virome, making up more than 1% each. Of these, seven can be linked to the *Podoviridae*, two to the *Siphoviridae* family, while five others correlated to unclassified dsDNA phages. Due to abundance corrections introduced by GAAS, the most abundant virotypes in terms of number of mapped reads were different from the most abundant ones selected based on GAAS-corrected values. The combined list of the most abundant phage sequences is given in Table 1. Of special notice is *Pelagibacter* phage HTVC010P [35], which made up 1.8% of the virome (GAAS-corrected value) with 4,223 reads mapped to its genome. Pelagiphages are possibly among the most numerous types of viruses on the planet [35], but little is known about their role in freshwater environments. One of the top 21 contigs in terms of the number of mapped reads assembled in this work (LNW4-c10) also had the TerL gene showing high similarity to the TerL of *Pelagibacter* phage HTVC010P (see below). Three other *Pelagibacter* phage sequences were identified in the Lough Neagh dataset, constituting 1.4% of the virome, with 5,527 reads mapped to their genomes. In agreement with the dominance of Cyanobacteria in the microbial community structure, 39,845 (9.00%) reads from the whole virome were annotated as originating from bacteriophages of *Synechococcus* and *Prochlorococcus* cyanobacteria as well as unclassified cyanophages.

**Table 1:** The most abundant virotypes in Lough Neagh virome^a^.

Phage	Accession number	Family	Genome size	Host	Abundance (GAAS-corrected, %)	Abundance (reads > 3000)
Salicola phage CGphi29	NC_020844.1	unclassified dsDNA phages	40695	*Salicola* sp. M5 MPN 10^-2 23B; Gammaproteobacteria	6.10	16293
Ralstonia phage RSK1	NC_022915.1	Podoviridae	40471	*Ralstonia solanacearum*; Betaproteobacteria	4.94	13132
Vibrio phage VvAW1	NC_020488.1	Podoviridae	38682	*Vibrio vulnificus*; Gammaproteobacteria	4.77	12117
Thalassomonas phage BA3	NC_009990.1	Podoviridae	37313	*Thalassomonas loyana* LMG 22536; Gammaproteobacteria	2.79	6824
Persicivirga phage P12024L	NC_018272.1	unclassified dsDNA phages/Siphoviridae	35652	*Persicivirga* sp. IMCC12024; Bacteroidetes	2.39	5583
Pelagibacter phage HTVC010P	NC_020481.1	Podoviridae	34892	*Pelagibacter ubique*; Alphaproteobacteria	1.84	4223
Bordetella phage BIP-1	NC_005809.1	Podoviridae	42638	*Bordetella bronchiseptica*; Betaproteobacteria	1.83	5123
Myxococcus phage Mx8	NC_003085.1	Podoviridae	49534	*Myxococcus xanthus* strain DK883; Deltaproteobacteria	1.59	5173
Cyanophage KBS-S-2A	NC_020854.1	unclassified dsDNA phages/Siphoviridae	40658	*Synechococcus* sp. WH7803; Cyanobacteria	1.27	3402
Cyanophage KBS-P-1A	NC_020865.1	unclassified dsDNA phages/Podoviridae	40048	*Synechococcus* sp. WH7803; Cyanobacteria	1.06	3178
Synechococcus phage S-CBS3	NC_015465.1	Siphoviridae	33004	*Synechococcus* sp. CB0202; Cyanobacteria	1.37	2961
Liberibacter phage SC1	NC_019549.1	Podoviridae	40048	*Candidatus* Liberibacter asiaticus UF506; Alphaproteobacteria	1.11	2907
Rhodococcus phage RRH1	NC_016651.1	Siphoviridae	14270	*Rhodococcus rhodochrous* str. Rrho39; Actinobacteria	1.08	1011
Persicivirga phage P12024S	NC_018271.1	unclassified dsDNA phages/Siphoviridae	35700	Persicivirga sp. IMCC12024; Bacteroidetes	1.06	2492
Puniceispirillum phage HMO-2011	NC_021864.1	Podoviridae	55282	“*Candidatus* Puniceispirillum marinum” strain IMCC1322; Alphaproteobacteria	0.95	3462
Rhizobium phage 16–3	NC_011103.1	Siphoviridae	60195	*Sinorhizobium meliloti* 41; Alphaproteobacteria	0.87	3438
Synechococcus phage S-CBS4	NC_016766.1	Siphoviridae	69420	*Synechococcus* sp. CB0101; Cyanobacteria	0.86	3909
Cellulophaga phage phi38:1	NC_021796.1	Podoviridae	72534	*Cellulophaga baltica* NN016038; Bacteroidetes	0.75	3589
Cronobacter phage vB_CsaM_GAP32	NC_019401.1	Myoviridae	358663	*Cronobacter sakazakii* HPB 3290; Gammaproteobacteria	0.13	3069

^a^ Virotype here is defined as the taxonomic affiliation of the best of all BLASTx matches (score ≥ 50) of a read with the RefSeq viral protein database.

Virotypes with more than 3,000 reads assigned or whose proportion in the virome taxonomic composition normalised by the genome lengths of the virotypes constituted 1% or more (GAAS correction) are shown.

#### MG-RAST analysis of unassembled reads

**Fig 4:**
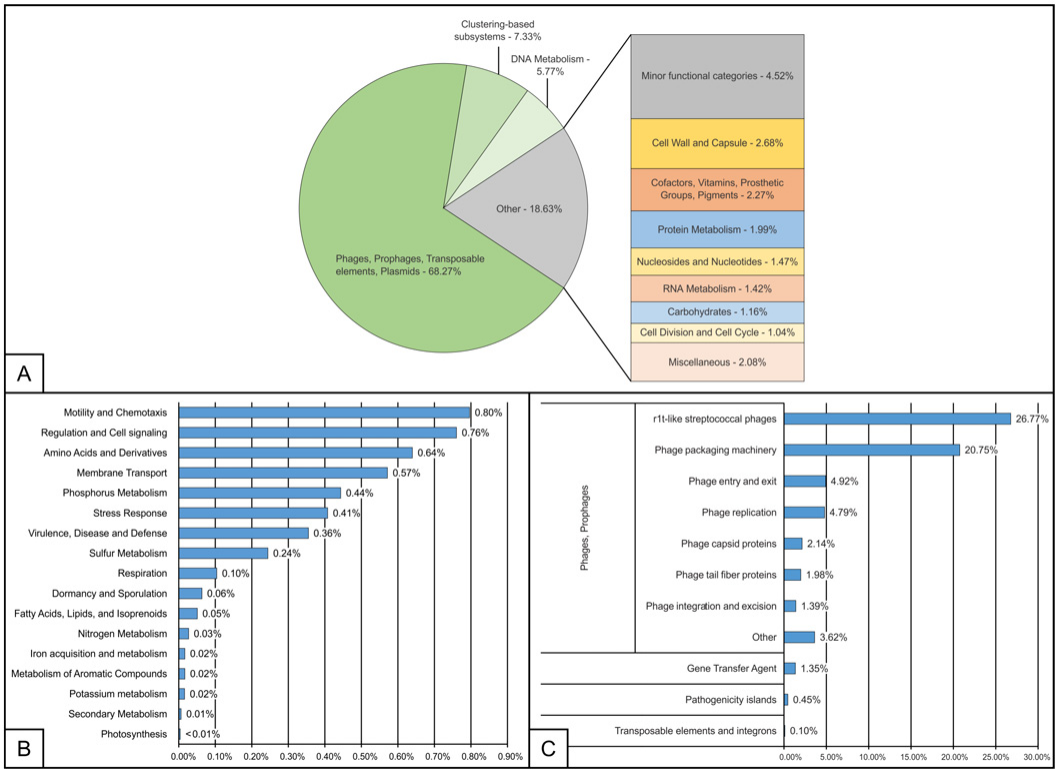
Functional analysis of Lough Neagh virome. The analysis was carried using SEED subsystems hierarchical functional annotation on the MG-RAST webserver. (A) Relative abundance of level one functional categories. (B) Distribution of minor functional categories. (C) Distribution of functional categories in the “Phages, Prophages, Transposable elements, Plasmids” group at levels 2 and 3.

After merging of paired-end reads, quality processing, and deduplication, the MG-RAST analysis pipeline [36] generated 2,601,470 reads. These reads were subjected to functional and taxonomic classification. MG-RAST utilises a number of different databases for functional annotation of reads, including four databases allowing for hierarchical functional annotation, namely KEGG Orthology (KO), COG, eggNOG, and SEED Subsystems [37]. The SEED subsystems database is manually curated and thus is considered to be more accurate. It is a conclusion reached by, for example, [37, 38], which we share, so we chose it as a primary method of functional annotation. The unassembled reads processed by MG-RAST were compared to the Subsystems database using a maximum e-value of 10^−5^, a minimum identity of 60%, and a minimum alignment length of 15 (measured in aa for protein and bp for RNA databases). 125,852 reads were classified this way. The functional distribution of reads at the highest hierarchical level of MG-RAST Subsystems classification is presented in Fig 4A. 68.3% of all classified reads were identified as belonging to the functional category of “Phages, Prophages, Transposable elements, and Plasmids”. Phages and prophages were the largest part of this group (66.4% of all classified reads), while 1.4% of reads belonged to the GTA (Gene Transfer Agents). A small number of reads in the functional category of “Phages, Prophages, Transposable elements, and Plasmids” were assigned to functional categories of”Pathogenicity islands” (0.5%) and “Transposable elements and integrons” (0.1%) (Fig 4C). It should be noted that in Fig 4C, in the category “Phages, Prophages” the top subgroup is “r1t-like streptococcal phages” (26.7%). We used functional classification based on SEED Subsystems. One of these subsystems, named “r1t-like streptococcal phages”, contains several genes characteristic of streptococcal bacteriophages, which are similar to phage r1t. The reads from our virome that had best BLAST hits to the genes in the category “r1t-like streptococcal phages” were classified as such, not necessarily originating from streptococcal phages. The remaining 21.7% reads were divided between various non-viral functional groups (Fig 4A). A detailed description of these groups presented in Fig 4B. It is important to note that the *pstS* (high affinity phosphate transporter) gene was identified in 116 reads. The *pstS* gene has previously been detected as integrated into genomes of a number of bacteriophages in a study of marine viruses by Sullivan and colleagues [39, 40]. To assess the extent of horizontal gene transfer we based the study of functional diversity of the virome on the analysis of individual reads, and not the assembled contigs. The presence of the *pstS* gene in our viral metagenome could arise from its being permanently integrated into a phage genome (specialized transducing phages) or from various transducing entities (generalised transducing phages or GTAs).

This study has produced the largest virome sequencing coverage of a freshwater lake to date. Nevertheless, the rarefaction analysis conducted clearly demonstrates that this sequencing is not exhaustive (S1A Fig). Comparison with previously published viromes of the French lakes Pavin and Bourget (S1B Fig), sequenced with less depth [12], demonstrated that the Lough Neagh virome has a higher sequence diversity. The lower limit of viral richness for Lough Neagh was estimated according to [41]. The average length of the 2,295,055 reads uploaded to MetaVir was 276 bp, and the reads were clustered into approximately 650,000 clusters at 90% identity level, and into approximately 840,000 clusters at 98% identity level. Using 50,000 bp as an average bacteriophage genome size, and defining “a single viral species” as in [41] (as being a grouping of isolates at nucleotide identity levels of 90% to 95%), we estimate the lower limit of the number of different viruses as being between 3588 and 4637, using the formula N*L/G, where N is the number of clusters, L the average read length (bp), and G the average bacteriophage genome size (bp). The Lough Neagh virome was also compared to freshwater viromes available on MetaVir (S2 Fig). Depending on the algorithm used for the comparison (di-, tri-, or tetranucleotide bias comparison [42] or BLAST-based comparison [32]), the closest viral communities identified were the viromes of Lagoa Vermelha [MetaVir project ID 4000], Tilapia_Channel– 1105 [MetaVir project ID 33] [11], El Berbera [MetaVir project ID 395] [9], and Lake Bourget [MetaVir project ID 7] [12], respectively.

According to MetaVir analysis, 14% of all reads were classified as of viral origin; the rest were not assigned. MG-RAST analysis of the same virome classified approximately 15% of the reads analysed. This means that over 80% of the sequences analysed lack any substantial homology to database entries (with an e-value smaller than 10^−5^). This is typical for those viral metagenomes analysed to date [41]. According to MG-RAST analysis, 10.9% of the reads were annotated as of bacterial origin (72% of all reads after QC and post-processing). This apparent anomaly could be explained by the fact that sequences of GTAs, bacterial vesicles, free external DNA, malformed VLPs (with bacterial DNA), and transduced bacterial DNA would be included in this category. It is also should be taken into account that the MG-RAST pipeline is heavily biased towards the annotation of sequences as being of bacterial origin. All precautions were taken in this work to minimise external bacterial DNA contamination; the VLP fraction was treated with an excess of DNase I as recommended [43] until disappearance of the 16S rRNA gene products (results not shown). Indeed, only 4 of 2,601,470 reads were classified as originating from 16S rRNA genes. These are likely to originate from general transducing phages or GTA particles.

When compared with two temperate freshwater viromes published [12], the striking difference is the absence of ssDNA viruses in Lough Neagh metagenome (0.5%); comparable values are 80% for Lake Pavin and 85% for Lake Bourget. The most likely explanation of this is the difference in preparation of the metagenomic samples for sequencing. No multiple displacement amplification (MDA), which is known to be highly biased towards the amplification of single-stranded DNA molecules [44, 45], was used in our work. In another viral metagenome project, where MDA was also not employed, ssDNA viruses also constituted less than 1% of all raw reads [41]. It may be concluded that avoiding the amplification of viral metagenomic samples using MDA is desirable for a more accurate representation of viral communities.

#### Contig construction and MetaVir analysis

66,450 contigs ranging from 301 to 58,805 bp were produced as described in the Experimental Procedure section. All contigs were uploaded to MetaVir server for annotation and comparison with other publicly available viromes. There were 21 contigs larger than 30 kb, with the largest being 58.8 kb. The essential characteristics of these contigs are presented in Table 2. The in-depth analysis has been conducted for largest contigs (i.e., LNW4-c0 –LNW4-c20), as well as for those which were detected as the most abundant in Lough Neagh (identified by high sequence coverage). As can be seen from the Table 2, putative cyanophages are highly represented in the Lough Neagh virome (contigs LNW4-c0, LNW4-c11, LNW4-c20).

**Table 2:** Analysis of contigs^a^ identified in the Lough Neagh virome.

Contig ID	Length (bp)/type	No reads/ total bases mapped/average coverage	No of ORF identified	No of ORF affiliated/genome percentage	Best BLAST hit affiliation		Terminase large subunit (TerL) affiliation	
					ID/score	Closest relative	ID/ score	Phage/Putative host (Genus; Phylum)
LNW4-c0	58073 /linear	2723/667258 /11.49	52	37/83.28	YP_009098966.1/602	Myoviridae; Caulobacter phage Cr30	YP_004324947.1/553	P-SSM7 / *Prochlorococcus*; Cyanobacteria
LNW4-c1	55834 /circular	5655/1258376 /22.54	73	19/35.29	YP_006383486.1/591	Podoviridae; Xylella phage Xfas53	YP_003344921.1/528	Xfas53 / *Xylella*; Gammaproteobacteria
LNW4-c2	54796 /circular	4669/1053524 /19.23	76	17/23.37	YP_224199.1/180	Siphoviridae Enterobacteria phage ES18	YP_006489258.1/86.7	9A / *Colwellia*; Gammaproteobacteria
LNW4-c3	40404 /linear	2894/662029 /16.39	60	11/22.35	YP_009031414.1/173	Myoviridae Bacillus phage Bcp1	YP_009099225.1/119	CP-51 / *Bacillus*; Firmicutes
LNW4-c4	38798 /linear	5661/1265474 /32.62	57	25/60.34	YP_007518354.1/405	Podoviridae Vibrio phage VvAW1	YP_007011072.1/306	SC1 / *Liberibacter*; Alphaproteobacteria
LNW4-c5	39931 /linear	3650/821026 /20.56	49	13/54.52	YP_239310.1/330	Caudovirales Xanthomonas phage Xp15	N/a	N/a
LNW4-c6	37802 /circular	4683/1092143 /28.89	54	20/52.19	YP_006560429.1/329	unclassified dsDNA phages Persicivirga phage P12024L	YP_008241822.1/218	phi46:1 / *Bacteroidetes*; Cellulophaga
LNW4-c7	37156 /linear	5693/1280891 /34.47	53	24/60.40	YP_007518354.1/387	Podoviridae Vibrio phage VvAW1	YP_007011113.1/336	SC2 / *Liberibacter*; Alphaproteobacteria
LNW4-c8	36891 /linear	6328/1428447 /38.72	36	6/43.74	YP_794082.1/225	Lambda-like viruses Stx2-converting phage 86	YP_009101181.1/89.7	9NA / *Salmonella*—Gammaproteobacteria
LNW4-c9	35854 /circular	9679/2177869 /60.74	65	18/42.14	YP_007518354.1/333	Podoviridae Vibrio phage VvAW1	YP_579204.1/122	mu1/6 / *Streptomyces*; Actinobacteria
LNW4-c10	35099 /circular	5110/1249796 /35.61	57	19/41.36	YP_006382527.1/394	Podoviridae Pseudomonas phage tf	YP_007517700.1/373	HTVC010P / *Pelagibacter*; Alphaproteobacteria
LNW4-c11	34867 /linear	2341/529557 /15.19	55	11/49.31	YP_001700553.1/211	Salmonella phage Fels-1	YP_004421530.1/197	S-CBS2 / *Synechococcus*; Cyanobacteria
LNW4-c12	34467 /circular	5954/1322394 /38.37	53	25/66.35	YP_009100954.1/706	Idiomarinaceae phage 1N2-2	YP_004421831.1/341	RDJL Phi 1 / *Roseobacter*; Alphaproteobacteria
LNW4-c13	34332 /linear	3257/739420 /21.54	56	19/43.84	YP_007518354.1/359	Podoviridae Vibrio phage VvAW1	YP_007675406.1/239	pCB2047-A / *Sulfitobacter*;—Alphaproteobacteria
LNW4-c14	34831 /linear	5882/1471014 /42.23	41	13/46.19	YP_008242145.1/518	Podoviridae Cellulophaga phage phi13:2	N/a	N/a
LNW4-c15	33144 /linear	2538/590177 /17.81	45	13/39.86	NP_203495.1/218	Podoviridae Myxococcus phage Mx8	YP_007111578.1/211	mEp235 / *Escherichia*; Gammaproteobacteria
LNW4-c16	33090 /linear	6484/1439188 /43.49	29	8/41.76	YP_005098041.1/188	Siphoviridae Pseudomonas phage phi297	N/a	N/a
LNW4-c17	31201 /linear	2797/701505 /22.48	50	7/21.35	YP_004421457.1/258	Siphoviridae Synechococcus phage S-CBS2	YP_002332340.1/81.3	MP38 / *Pseudomonas*; Gammaproteobacteria
LNW4-c18	31150 /linear	2094/466073 /14.96	43	17/39.57	YP_223952.1/395	Siphoviridae Yualikevirus Phage phiJL001	N/a	N/a
LNW4-c19	30597 /linear	8519/2021986 /66.08	55	14/34.82	YP_006906634.1/182	Siphoviridae Salmonella phage SSU5	YP_006906634.1/182	SSU5 / *Salmonella*; Gammaproteobacteria
LNW4-c20	58805 /linear	4750/1068733 /18.17	67	22/46.54	YP_239310.1/329	Caudovirales Xanthomonas phage Xp15	YP_007006119.1/198	S-TIM5 / *Synechococcus*; Cyanobacteria

^a^ Only contigs with the length over 30,000 bp are presented.

N/a, no terminase large subunit (TerL) gene found.

**Fig 5:**
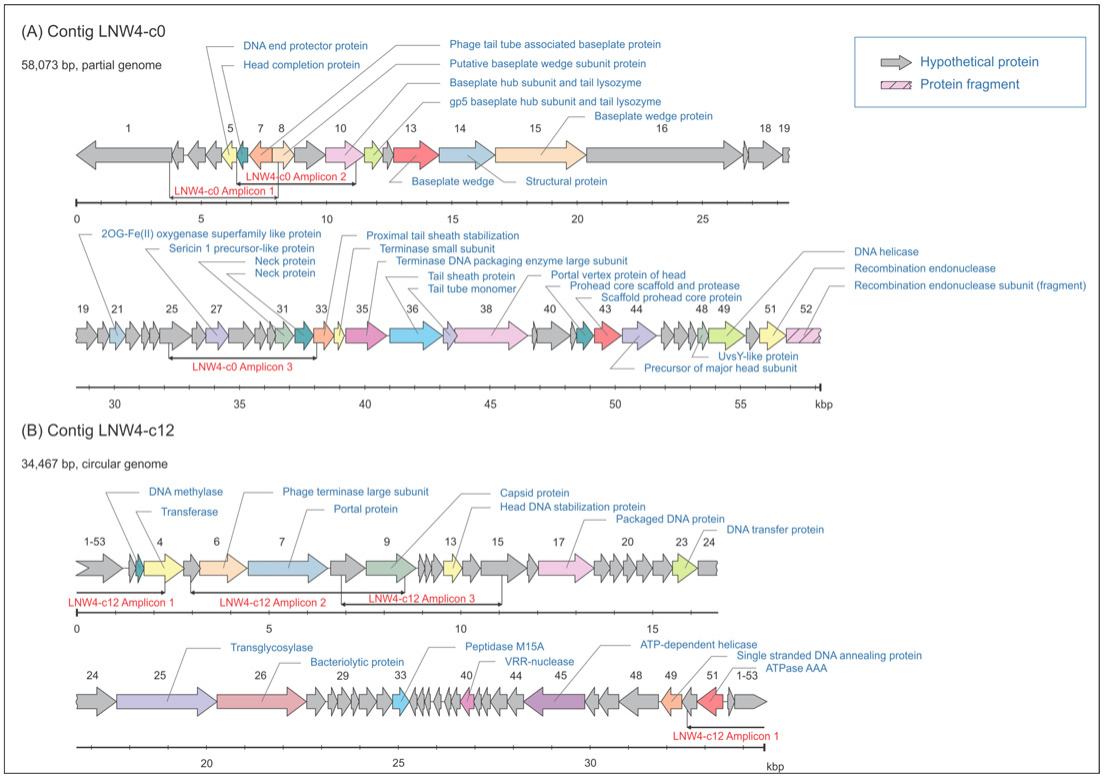
Maps of putative phage genomes identified in Lough Neagh. Genome regions amplified using PCR and genome specific primers are indicated with horizontal bars. Identified ORF shown by arrows. (A) Genome map of putative phage LNW4-c0. (B) Genome map of putative phage LNW4-c12.

Genetic maps for contigs LNW4-c0 and LNW4-c12 are shown in Fig 5. LNW4-c0 represents a putative *Myoviridae* (possibly T4-like) phage. 51 full and 1 partial ORFs were identified in this contig of 58,073 bp. On the basis of the analysis of orf35, identified as a terminase large subunit by BLASTp and hmmscan comparisons, this phage can be classified as being related to *Prochlorococcus* phage P-SSM7 (NC_015290.1) and *Sinorhizobium* phage phiM12 (KF381361). While it is impossible to unambiguously determine the taxonomic affiliation of the phage in question, the similarity of a number of other ORFs of the contig to genes of cyanophages favours the hypothesis of a cyanophage origin. The genome sizes of both related phages are more than 150 kb; therefore, it is likely that LNW4-c0 contig represents a partial sequence of a phage genome from Lough Neagh. LNW4-c12 probably comes from a member of *Podoviridae* family, this 34,467 bp circular contig contains 52 ORFs. It is likely that this contig represents a genome of a phage with either circular permutations or long direct terminal repeats. According to MetaVir BLASTp and independent BLASTx analyses, the closest homologs of the LNW4-c12 TerL gene are sequences of the terminase large subunit from *Roseobacter* phage RDJL Phi 1 (62,668 bp) and the terminase large subunit from the *Burkholderia* sp. TJI49 phage genome, respectively. Due to a high diversity of environmental bacteriophages and a limited number of viral genomes available in the reference databases, it is not possible to state whether or not LNW4-c12 is indeed a phage infecting bacteria of genus *Roseobacter* or *Burkholderia*.

To confirm that the identified contigs LNW4-c0 and LNW4-c12 corresponded to the genomic DNA molecules present in the sample analysed, three pairs of specific primers were designed for each of these two contigs to amplify segments 4–6 kbp long, and PCR reactions were performed using the same metagenomic DNA that had been used for Illumina sequencing. In all six cases, PCR products were obtained and Sanger sequencing analysis confirmed the presence of these contigs (the PCR amplified and confirmed regions are indicated in Fig 5).

## Conclusions

Lough Neagh is the largest and the most important freshwater lake of the British Isles. Here for the first time, a metagenomic analysis of the microbial community of the lake has been conducted with an emphasis on characterisation of the virome. As in the majority of previously characterised viromes a large number (85%) of the reads did not have homologs in available databases. However, this work demonstrates that the microbial community of Lough Neagh is clearly different from those of major freshwater lakes previously analysed. The most important of these differences are: *i*) the abundance of Cyanobacteria (27%) and paucity of Actinobacteria; *ii*) the apparent abundance of putative cyanophages in the Lough Neagh virome; *iii*) the high diversity of the virome. The abundance of the Cyanobacteria group is most likely a result of intensive agricultural activity in the area leading to ecological damage to this freshwater system [29]. It is difficult to reliably assess the proportion of phages infecting Cyanobacteria in Lough Neagh due to the absence of universal genetic markers for this group of viruses (and for bacteriophages in general). However, we were able to identify a number of putative cyanophage genomes (Tables 1 and 2) abundant in the Lough Neagh ecosystem. The assembled contig of phage LNW4-c0 was confirmed in PCR experiments using the corresponding metagenomic DNA. Previous works on the viral communities from the marine environments provide valuable information about the role of cyanophages. For example, earlier studies by Paul and colleagues, who investigated bacteria-phage relationships in the marine environment, indicated that an environment inimical to bacterial growth supports lysogeny [46, 47]. Studies of the marine cyanobacterium *Synechococcus* indicated that phage S-PM2 infecting this species preferentially enters into a lysogenic state in phosphate (Pi)-depleted waters [48, 49]. A study of various phages infecting Cyanobacteria in the marine environment identified phage encoded genes for alkaline phosphatase (*phoA*) and the periplasmic high affinity phosphate-binding protein (*pstS*) [50]. Crucially, the transcriptional activity of these was shown to be activated in Pi-starved bacteria and controlled by the host’s Pi starvation response regulon [51]. It is likely that Pi levels play an important role in phage production in the marine environment and, importantly, that the corresponding phages could serve as early indicators of the phosphate status of the environment. While relatively little is known about freshwater cyanophages, it is important to note that identified cyanophage-derived contigs (Table 1, Table 2, and contig annotations and phylogenetic trees available on the Metavir website in the project Lough Neagh—4pW contigs, Project id: 5053), suggest the ubiquity and importance of cyanophages in the Lough Neagh freshwater ecosystem, where they might play roles similar to those of marine phages. It is important to note that the *pstS* gene, which was shown to be integrated into genome of some marine cyanophages, was found to be present in the Lough Neagh metagenome. This may indicate the horizontal gene transfer of this gene by generalised transducing cyanophages or GTAs in a freshwater environment.

## Experimental Procedures

All prevailing local, national, and international regulations and conventions, and normal scientific ethical practices have been respected. No specific approvals and permissions were required to collect and process water samples from Lough Neagh, as all the work conducted did not involve endangered or protected species and was carried on outside of privately owned or protected areas.

### Primary water sample

Lough Neagh (54°37′06″N, 6°23′43″W) is the largest lake in the British Isles. Three 10 m integrated water column samples of 5 litres each were collected from Lough Neagh using a flexible hose at a site situated approximately 5 km North from Kinnego Marina on 28 April 2014 at 11:00 GMT, taken to the laboratory within 2 hours and placed on ice. The Secchi depth, temperature and pH of lake water at surface, 5 m and 10 m depths were recorded on the site and several extra water samples were taken for chemical analysis (S1 Table).

### Additional water samples

Five additional water column samples were collected from Lough Neagh at the same location as the primary sample on 1 April 2014, 23 June 2014, 2 September 2014, 10 November 2014, and 18 February 2015. These water samples were processed in the same manner as the primary sample and were used for the analysis of taxonomic composition of bacterial communities via 16S rRNA gene amplicon sequencing.

### Primary sample processing and DNA extraction

The samples were processed within 24 h of collection. Total DNA was extracted from 500 ml of water using sterile 0.2 μm ME 24 ST Mixed Cellulose Ester Membrane filters (Whatman/GE Healthcare, UK) and PowerWater DNA Isolation kit (MO BIO, USA). To obtain a ‘virus-like particle’ (VLP) fraction, 5 litres of water were filtered through 0.22 μm Steripak GP-20 filter units (EMD Millipore, USA) and concentrated to 50 ml using an LV Centramate Lab Tangential Flow Filtration System with a 100 kDa Omega membrane suspended screen cassette (Pall, USA). To ensure removal of any remaining planktonic microorganisms, the preparation was further filtered through 0.22 μm Millex-GS syringe filter units (EMD Millipore, USA). The filtrate was concentrated into a final volume of 4 ml using an Amicon Ultra-15 Centrifugal Filter Unit with 100-kDa molecular mass cut-off (EMD Millipore, USA). The resulting VLP concentrate was incubated with 3,000 U of DNase I (Roche, USA) at 4°C for 24 h. PCR with universal 16S rRNA gene primers (63-F/1387-R) [52] was then carried out to confirm the removal of external bacterial DNA. DNase I treatment was continued until no 16S rRNA gene sequences could be detected in the sample. Viral DNA was isolated from the purified VLP concentrate by a formamide/CTAB extraction procedure [53, 54], purified with PowerClean Pro DNA Clean-Up Kit (MO BIO, USA) and quantified using a Quantus fluorometer (Promega, USA). The absence of bacterial contamination was monitored at all stages by epifluorescence microscopy of the SYBR Gold (Invitrogen, USA) stained samples as previously described [55].

### Preparation of libraries and sequencing

A 16S rRNA gene amplicon library was constructed from total DNA of the primary sample. Partial bacterial 16S rRNA gene sequences were amplified from the total DNA sample by two-step PCR with primers 909-F/1492-R (1^st^ step, 27 cycles) and 909-F B Lib L/1492-Tag 4 A Lib L (2^nd^ step, 5 cycles) [56, 57]. The primers 909-F/1492-R used for the first step of amplification were evaluated in the study by Klindworth et al. [58] and demonstrated good coverage of the domain Bacteria (specifically, if no mismatches are allowed, 91.7% by primer 909-F and 73.4% by primer 1492-R). The resulting PCR amplicons were purified with a High Pure PCR Product Purification Kit (Roche, USA) and quantified using a Quantus fluorometer (Promega, USA). Amplicon sequencing was performed on a 454 GS Junior (Roche, USA) with Lib-L Shotgun chemistry at the University of Cambridge DNA Sequencing Facility.

Viral DNA of the primary sample was subjected to whole genome shotgun (WGS) sequencing at the University of Cambridge DNA Sequencing Facility. A Nextera DNA Sample Preparation kit (Illumina, USA) was used to generate the sequencing library directly from 50 ng of metagenomic viral DNA without preliminary amplification. A 1% PhiX v3 library spike-in was used as a quality control for cluster generation and sequencing. The resulting library was sequenced from both ends (2×300 bp) with the 600-cycle MiSeq Reagent Kit v3 on MiSeq (Illumina, USA). Sequencing adaptors were trimmed off the raw reads at the sequencing facility.

### Primary sample bacterial community analysis

The raw reads obtained from sequencing of total water column 16S rRNA gene amplicons were processed using the QIIME pipeline v 1.8.0 [14], following standard protocols. Briefly, the reads were length- and quality-filtered and de-noised, yielding 3,275 sequences for downstream analyses (S1 Datasets, 28_April_2014). Operational taxonomic units (OTUs) were picked using the usearch clustering and quality-filtering method with default parameters. OTUs were clustered at the sequence similarity level of 97%, with a minimum cluster size of 4. The detection and discarding of chimeric sequences was performed by usearch, using both *de novo* and reference-based detection (ChimeraSlayer reference database, version microbiomeutil-r20110519). The most abundant sequences found in the OTUs were selected as representative sequences. Taxonomic assignment of OTUs was performed using the RDP classifier and Greengenes reference database v 13.8, with minimum confidence score of 0.5. Unclassified and chloroplast-related sequences were filtered out from the OTU table. Statistical analyses were performed using R and GraphPad Prism.

### Primary sample virome processing and analysis

Illumina sequencing of viral DNA produced 2,298,791 2×300 bp reads. The reads obtained ranged from 35 to 300 bp in length, with an average length of 263 bp, and a median length of 299 bp. The sequence data files have been submitted to NCBI Sequence Read Archive (SRA, http://www.ncbi.nlm.nih.gov/sra) under the following accession numbers: SRP062094 (study), SRR2147000 (sequencing run). Initial quality control was performed with FastQC (http://www.bioinformatics.babraham.ac.uk/projects/fastqc/) and the NGS QC Toolkit [59], and reads were processed with BBMap v 33.54 (http://sourceforge.net/projects/bbmap/). Briefly, all reads with an average Q-score < 13 or containing Ns were discarded. The reads were then trimmed of adaptors and quality-trimmed (trimq = 15) by bbduk.sh script. Finally, bbmerge.sh was used to merge paired-end reads having an overlap of at least 20 bp, and all reads shorter than 30 bp were discarded.

The IDBA-UD sequence assembler v1.1.1 [60] was used to assemble the processed reads into contigs. Two modifications were made to the source code before compiling: in the source code file idba-1.1.1\src\basic\kmer.h the expression "static const uint32_t kNumUint64 = 4" was changed to "static const uint32_t kNumUint64 = 16"; in the source code file idba-1.1.1\src\sequence\short_sequence.h the expression "static const uint32_t kMaxShortSequence = 128" was changed to "static const uint32_t kMaxShortSequence = 32768". The assembly was performed using the following parameters:—mink 20—maxk 250—step 20—num_threads 8. Reads were mapped to contigs with Bowtie2 [61] and mapping statistics were obtained using SAMtools [62] and BEDTools [63], while Artemis [64] and IGV [65] were used for mapping visualisation. The virome was analysed using two online pipelines: MetaVir and MG-RAST. The contigs and unassembled processed reads were uploaded to the MetaVir [33] (http://metavir-meb.univ-bpclermont.fr/) server for taxonomic annotation and comparison with other publicly available viromes (project ID 4925 –Lough Neagh virome, project ID 5053 –Lough Neagh assembled contigs). Functional annotation of the virome was performed with MG-RAST [36] (http://metagenomics.anl.gov/). Due to the fact that MG-RAST implements its own quality-filtering and pre-processing pipeline, the original unprocessed reads were uploaded (MG-RAST ID 4585272.3).

## Supporting Information

S1 Datasets 16S rRNA gene amplicons from the Lough Neagh water samples. (ZIP)

S1 Fig Rarefaction analysis of Lough Neagh virome. (A) A rarefaction curve of the total viral metagenome was obtained after high-throughput sequencing of the Lough Neagh sample. The rarefaction curve was constructed within MetaVir with clustering set at 90% identity; 2,295,055 reads were analysed. (B) Comparison of the rarefaction curves of the three freshwater viral metagenomes conducted using MetaVir; subsamples of 50,000 reads from each virome were used. Red, Lough Neagh; green, Lake Pavin; blue, Lake Bourget.
(TIF)

S2 Fig Score matrices-based global comparisons of Lough Neagh virome to freshwater viromes at MetaVir website. Results of oligonucleotide signatures comparison of full viromes and BLAST-based comparison of 50,000 sequences are shown. Hierarchical clustering and tree generation were done by R package pvclust. (A) Dinucleotide composition bias comparison. (B) Trinucleotide composition bias comparison. (C) Tetranucleotide composition bias comparison. (D) BLAST-based comparison.
(PDF)

S1 File Comparison of bacterial communities of Lough Neagh and selected freshwater lakes. (PDF)

S1 Table Chemical and environmental parameters of the Lough Neagh water sample used for metagenomic analysis. (XLSX)
