# Supplementary figures and images for: Metagenomic Characterisation of the Viral Community of Lough Neagh, the Largest Freshwater Lake in Ireland
Source: PLoS One. 2016 Feb 29;11(2):e0150361. doi: 10.1371/journal.pone.0150361 (PMC4771703; doi:10.1371/journal.pone.0150361)

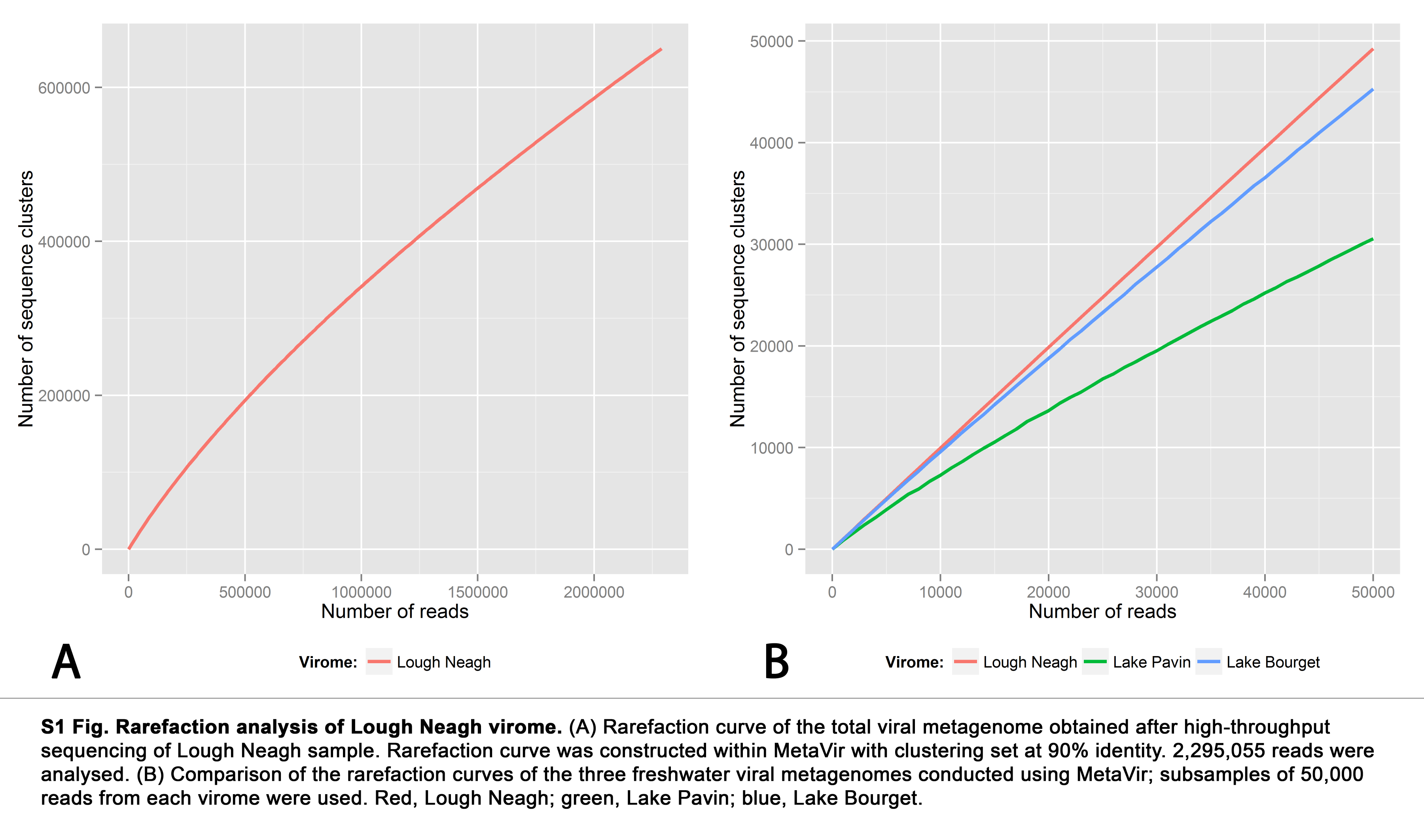

Supplement: S1 Fig — (A) A rarefaction curve of the total viral metagenome was obtained after high-throughput sequencing of the Lough Neagh sample. The rarefaction curve was constructed within MetaVir with clustering set at 90% identity; 2,295,055 reads were analysed. (B) Comparison of the rarefaction curves of the three freshwater viral metagenomes conducted using MetaVir; subsamples of 50,000 reads from each virome were used. Red, Lough Neagh; green, Lake Pavin; blue, Lake Bourget. (TIF) [file pone.0150361.s002.tif]
