# Supplementary material for: Metagenomic Characterisation of the Viral Community of Lough Neagh, the Largest Freshwater Lake in Ireland
Source: PLoS One. 2016 Feb 29;11(2):e0150361. doi: 10.1371/journal.pone.0150361 (PMC4771703; doi:10.1371/journal.pone.0150361)
Supplement: S2 Fig — Results of oligonucleotide signatures comparison of full viromes and BLAST-based comparison of 50,000 sequences are shown. Hierarchical clustering and tree generation were done by R package pvclust. (A) Dinucleotide composition bias comparison. (B) Trinucleotide composition bias comparison. (C) Tetranucleotide composition bias comparison. (D) BLAST-based comparison. (PDF) [file pone.0150361.s003.pdf]

S2 Fig. Score matrices-based global comparisons of Lough Neagh virome to freshwater viromes at MetaVir website. Results of oligonucleotide signatures comparison of full viromes and BLAST-based comparison of 50,000 sequences are shown. Hierarchical clustering and tree generation were done by R package pvclust. (A) Dinucleotide composition bias comparison. (B) Trinucleotide composition bias comparison. (C) Tetranucleotide composition bias comparison. (D) BLAST-based comparison.

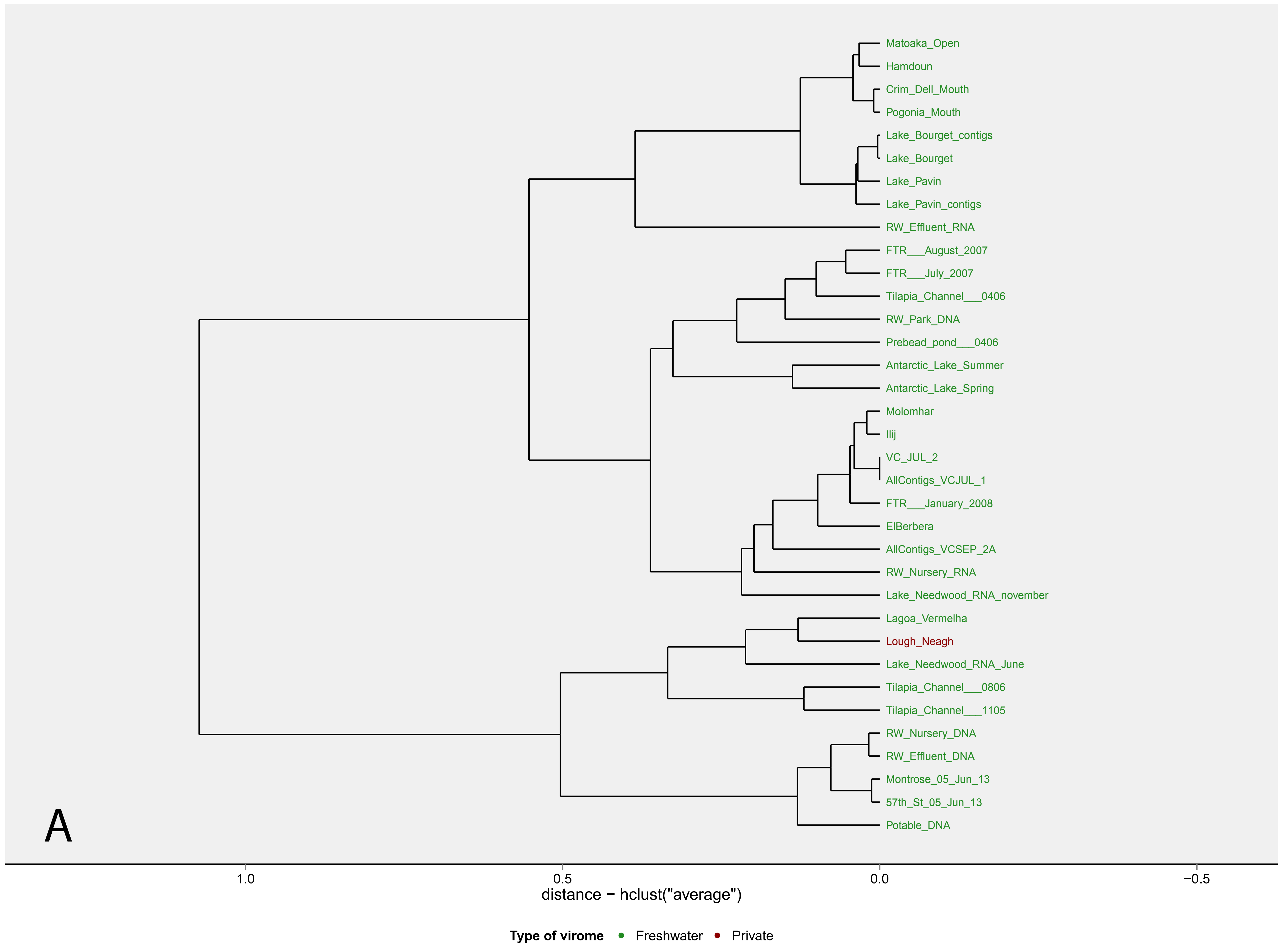
Matoaka_Open
Hamdoun
Crim_Dell_Mouth
Pogonia_Mouth
Lake_Bourget_contigs
Lake_Bourget
Lake_Pavin
Lake_Pavin_contigs
RW_Effluent_RNA
FTR___August_2007
FTR___July_2007
Tilapia_Channel___0406
RW_Park_DNA
Prebead_pond___0406
Antarctic_Lake_Summer
Antarctic_Lake_Spring
Molomhar
Ilij
VC_JUL_2
AllContigs_VCJUL_1
FTR___January_2008
ElBerbera
AllContigs_VCSEP_2A
RW_Nursery_RNA
Lake_Needwood_RNA_november
Lagoa_Vermelha
Lough_Neagh
Lake_Needwood_RNA_June
Tilapia_Channel___0806
Tilapia_Channel___1105
RW_Nursery_DNA
RW_Effluent_DNA
Montrose_05_Jun_13
57th_St_05_Jun_13
Potable_DNA
A
1.0
0.5
0.0
−0.5
distance − hclust("average")
Type of virome
Freshwater
Private

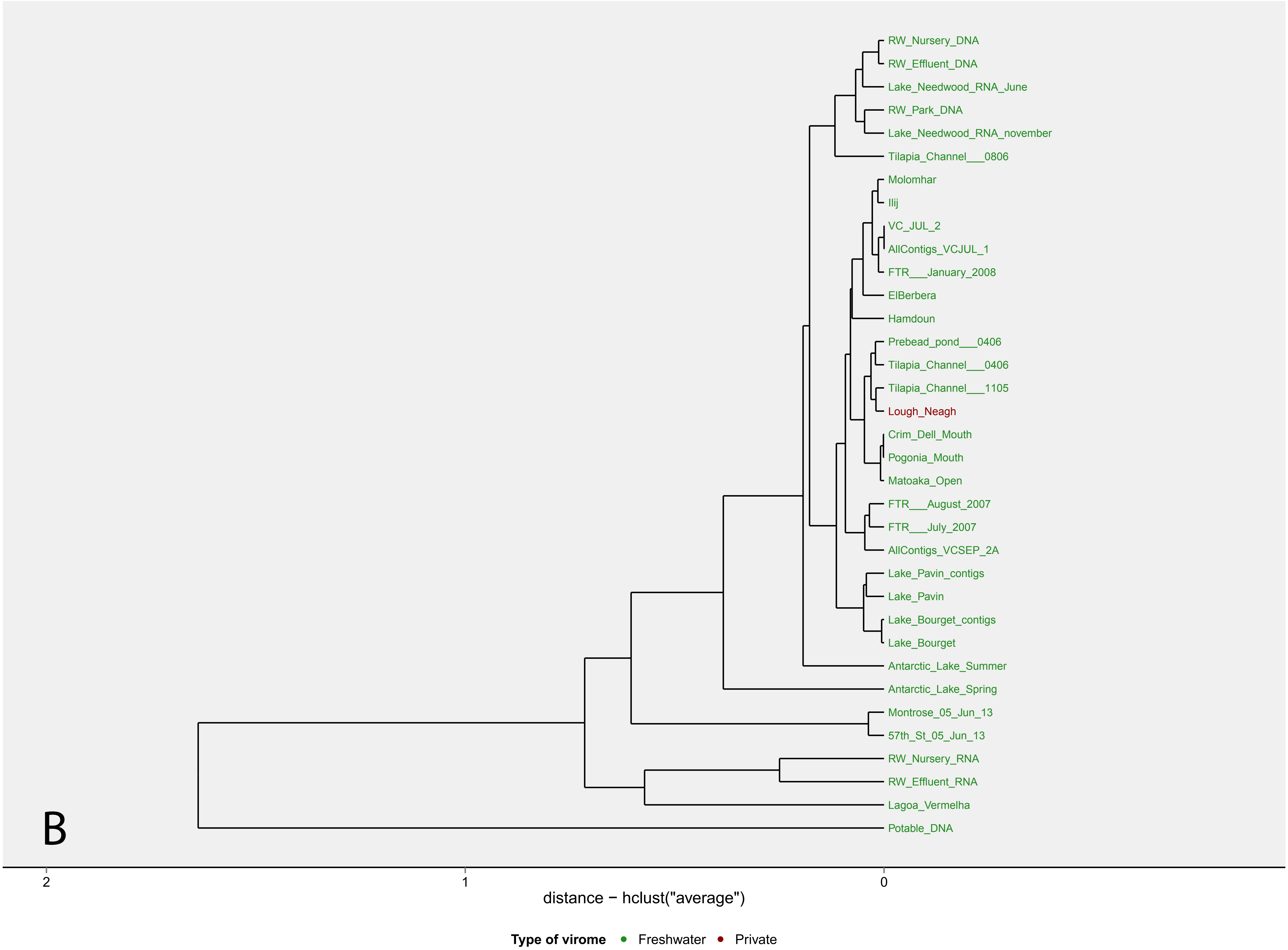
RW_Nursery_DNA
RW_Effluent_DNA
Lake_Needwood_RNA_June
RW_Park_DNA
Lake_Needwood_RNA_november
Tilapia_Channel___0806
Molomhar
Ilij
VC_JUL_2
AllContigs_VCJUL_1
FTR___January_2008
ElBerbera
Hamdoun
Prebead_pond___0406
Tilapia_Channel___0406
Tilapia_Channel___1105
Lough_Neagh
Crim_Dell_Mouth
Pogonia_Mouth
Matoaka_Open
FTR___August_2007
FTR___July_2007
AllContigs_VCSEP_2A
Lake_Pavin_contigs
Lake_Pavin
Lake_Bourget_contigs
Lake_Bourget
Antarctic_Lake_Summer
Antarctic_Lake_Spring
Montrose_05_Jun_13
57th_St_05_Jun_13
RW_Nursery_RNA
RW_Effluent_RNA
Lagoa_Vermelha
Potable_DNA
B
2
1
0
distance − hclust("average")
Type of virome
Freshwater
Private

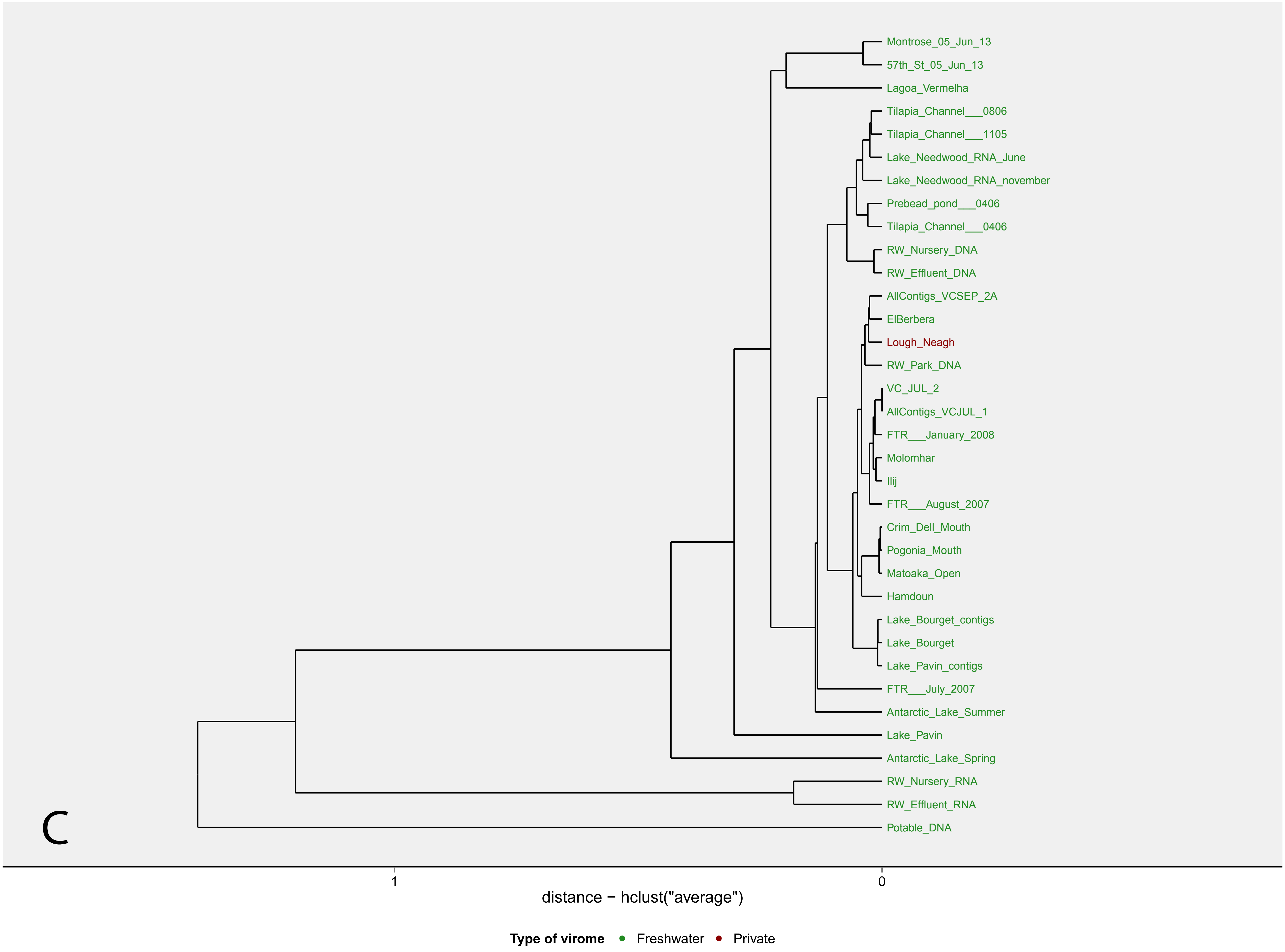
Montrose_05_Jun_13
57th_St_05_Jun_13
Lagoa_Vermelha
Tilapia_Channel___0806
Tilapia_Channel___1105
Lake_Needwood_RNA_June
Lake_Needwood_RNA_november
Prebead_pond___0406
Tilapia_Channel___0406
RW_Nursery_DNA
RW_Effluent_DNA
AllContigs_VCSEP_2A
ElBerbera
Lough_Neagh
RW_Park_DNA
VC_JUL_2
AllContigs_VCJUL_1
FTR___January_2008
Molomhar
Ilij
FTR___August_2007
Crim_Dell_Mouth
Pogonia_Mouth
Matoaka_Open
Hamdoun
Lake_Bourget_contigs
Lake_Bourget
Lake_Pavin_contigs
FTR___July_2007
Antarctic_Lake_Summer
Lake_Pavin
Antarctic_Lake_Spring
RW_Nursery_RNA
RW_Effluent_RNA
Potable_DNA
C
1
0
distance − hclust("average")
Type of virome
Freshwater
Private

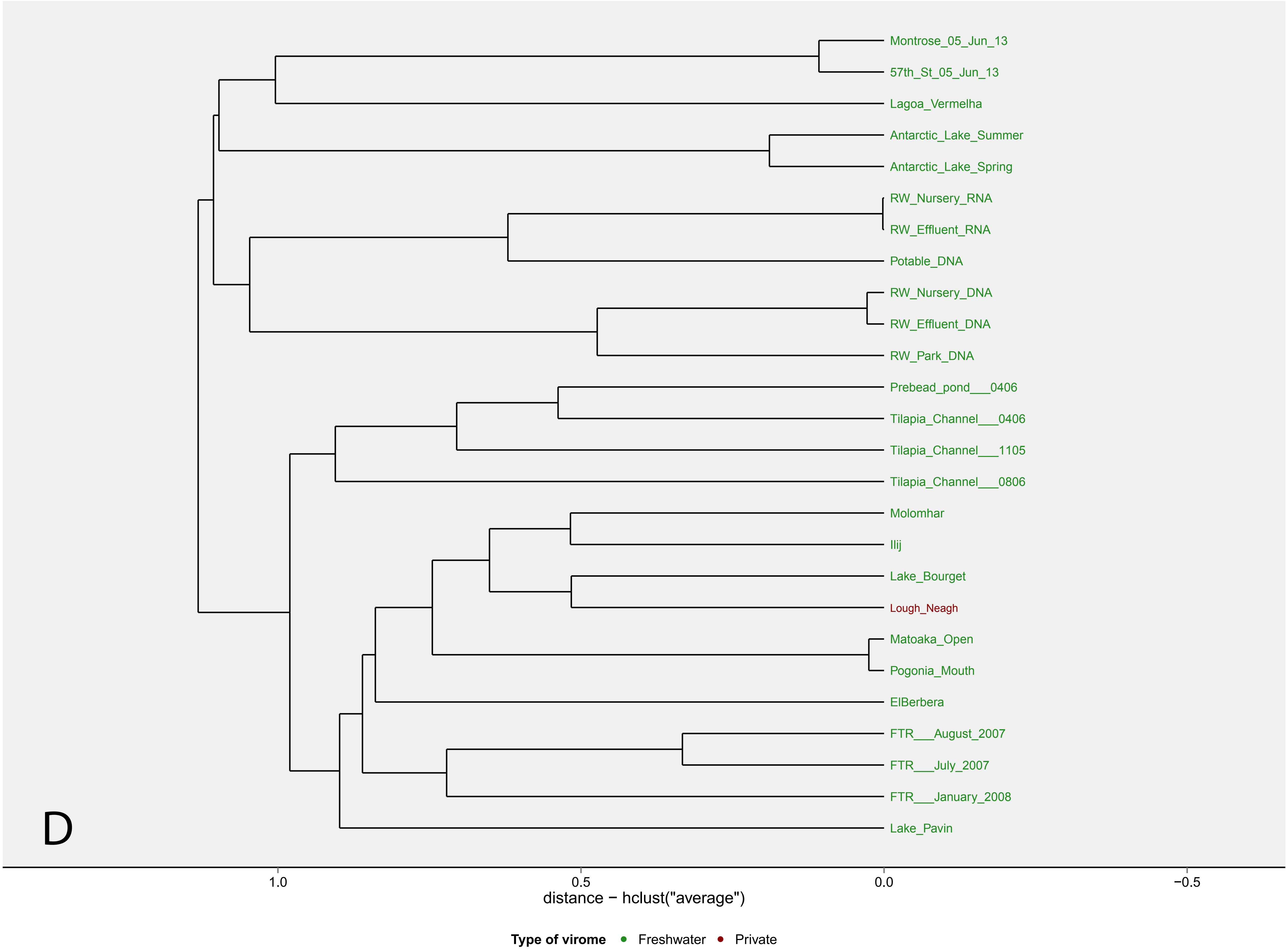
Montrose_05_Jun_13
57th_St_05_Jun_13
Lagoa_Vermelha
Antarctic_Lake_Summer
Antarctic_Lake_Spring
RW_Nursery_RNA
RW_Effluent_RNA
Potable_DNA
RW_Nursery_DNA
RW_Effluent_DNA
RW_Park_DNA
Prebead_pond___0406
Tilapia_Channel___0406
Tilapia_Channel___1105
Tilapia_Channel___0806
Molomhar
Ilij
Lake_Bourget
Lough_Neagh
Matoaka_Open
Pogonia_Mouth
ElBerbera
FTR___August_2007
FTR___July_2007
FTR___January_2008
Lake_Pavin
D
1.0
0.5
0.0
−0.5
distance − hclust("average")
Type of virome
Freshwater
Private
