# Supplementary material for: Metagenomic Characterisation of the Viral Community of Lough Neagh, the Largest Freshwater Lake in Ireland
Source: PLoS One. 2016 Feb 29;11(2):e0150361. doi: 10.1371/journal.pone.0150361 (PMC4771703; doi:10.1371/journal.pone.0150361)
Supplement: S1 File — (PDF) [file pone.0150361.s004.pdf]

S1 File. Comparison of bacterial communities of Lough Neagh and selected freshwater lakes. (A) The names of the selected freshwater lakes and the references of the corresponding studies. (B) Comparison of major bacterial phyla abundances in the selected freshwater lakes. 16S rRNA gene amplicon datasets were downloaded from GenBank and annotated taxonomically using QIIME pipeline. (C) Principal Coordinates Analysis. A table with the abundances of different bacteria in the compared lakes at the class level was generated and dissimilarity matrix was calculated using “Manhattan” method. The percentages of explained variation were calculated from eigenvalues and added to the X and Y axes. The analysis was performed in R version 3.2.2.

A

Name	Location	Coordinates	Climate zone	Trophic status	Maximum depth, m	Reference	Notes
Pavin	Europe, France	45°29′N; 2°56′E	Temperate	Oligomesotrophic	92	[1]	
Reservoir	Europe, France	46°2′N; 3°1′E	Temperate	Oligomesotrophic	37	[1]	
Annecy	Europe, France	45°52′N; 6°09′E	Temperate	Oligotrophic	65	[2]	Dataset Annecy Spr
Bagre	West Africa, Burkina Faso	11°29′N; 0°32′W	Tropical	—[a]	9.7	[2]	
Bamsa	West Africa, Burkina Faso	12°15′N; 1°06′W	Tropical	—[a]	3.1	[2]	
Bazega	West Africa, Burkina Faso	11°44′N; 1°21′W	Tropical	—[a]	3.2	[2]	
Bourget	Europe, France	45°42′N; 5°52′E	Temperate	Mesotrophic	145	[2]	Dataset Bourget Spr
Bourget 2	Europe, France	45°42′N; 5°52′E	Temperate	Mesotrophic	145	[3]	Dataset metagen16S_cs
Dem	West Africa, Burkina Faso	13°11′N; 1°09′W	Tropical	—[a]	1.5	[2]	
Ouagadougou	West Africa, Burkina Faso	12°23′N; 1°30′W	Tropical	—[a]	1.6	[2]	Combined datasets Ouaga 1 and Ouaga 2
Pouytenga	West Africa, Burkina Faso	12°12′N; 0°25′W	Tropical	—[a]	0.7	[2]	
Michigan	North America, USA	43°04.588N; 87°50.311W	Temperate	Oligotrophic	281	[4]	Linnwood 9m dataset
Lough Neagh	Europe, Northern Ireland	54°37′06″N, 6°23′43″W	Temperate	Hypertrophic	25	This study	28 April 2015 dataset

[a] While the trophic status for Burkina Faso lakes wasn't explicitly indicated in the original publication, it ranged (according to the authors of the original paper) from oligotrophic to eutrophic.

References

1. Boucher D, Jardillier L, Debroas D. Succession of bacterial community composition over two consecutive years in two aquatic systems: a natural lake and a lake-reservoir. FEMS Microbiol Ecol. 2006;55:79–97.
2. Humbert JF, Dorigo U, Cecchi P, Berre BL, Debroas D, Bouvy M. Comparison of the structure and composition of bacterial communities from temperate and tropical freshwater ecosystems. Environmental Microbiology. 2009;11(9):2339–2350.
3. Debroas D, Humbert JF, Enault F, Bronner G, Faubladier M, Cornillot E. Metagenomic approach studying the taxonomic and functional diversity of the bacterial community in a mesotrophic lake (Lac du Bourget--France). Environmental microbiology. 2009;11(9):2412-24.
4. Mueller-Spitz SR, Goetz GW, McLellan SL. Temporal and spatial variability in nearshore bacterioplankton communities of Lake Michigan. FEMS Microbiol Ecol. 2009;67:511–522.

B

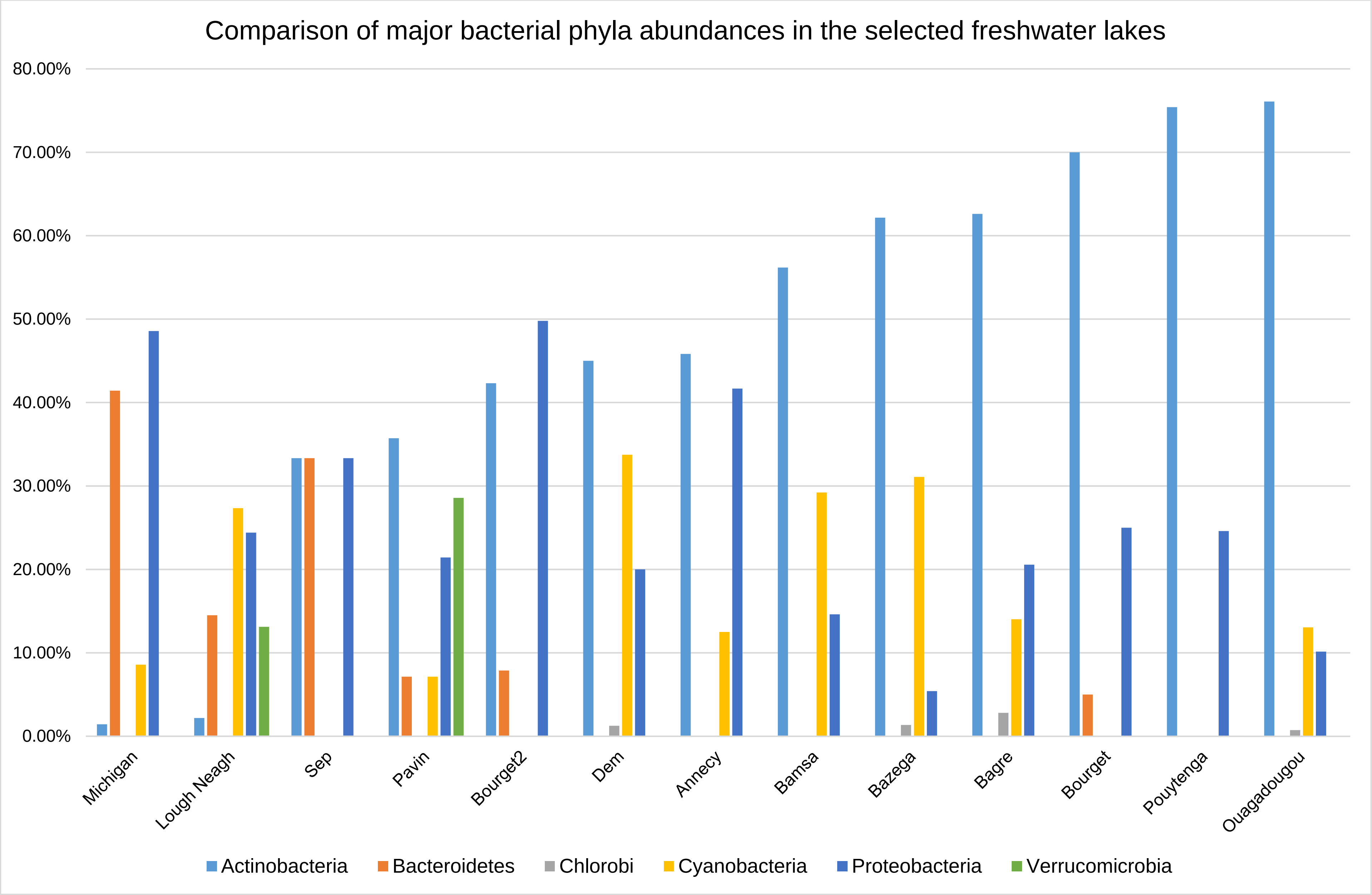

Comparison of major bacterial phyla abundances in the selected freshwater lakes
80.00%
70.00%
60.00%
50.00%
40.00%
30.00%
20.00%
10.00%
0.00%
Michigan
Lough Neagh
Sep
Pavin
Bourget2
Dem
Annecy
Bamsa
Bazega
Bagre
Bourget
Pouytenga
Ouagadougou
Actinobacteria
Bacteroidetes
Chlorobi
Cyanobacteria
Proteobacteria
Verrucomicrobia

C

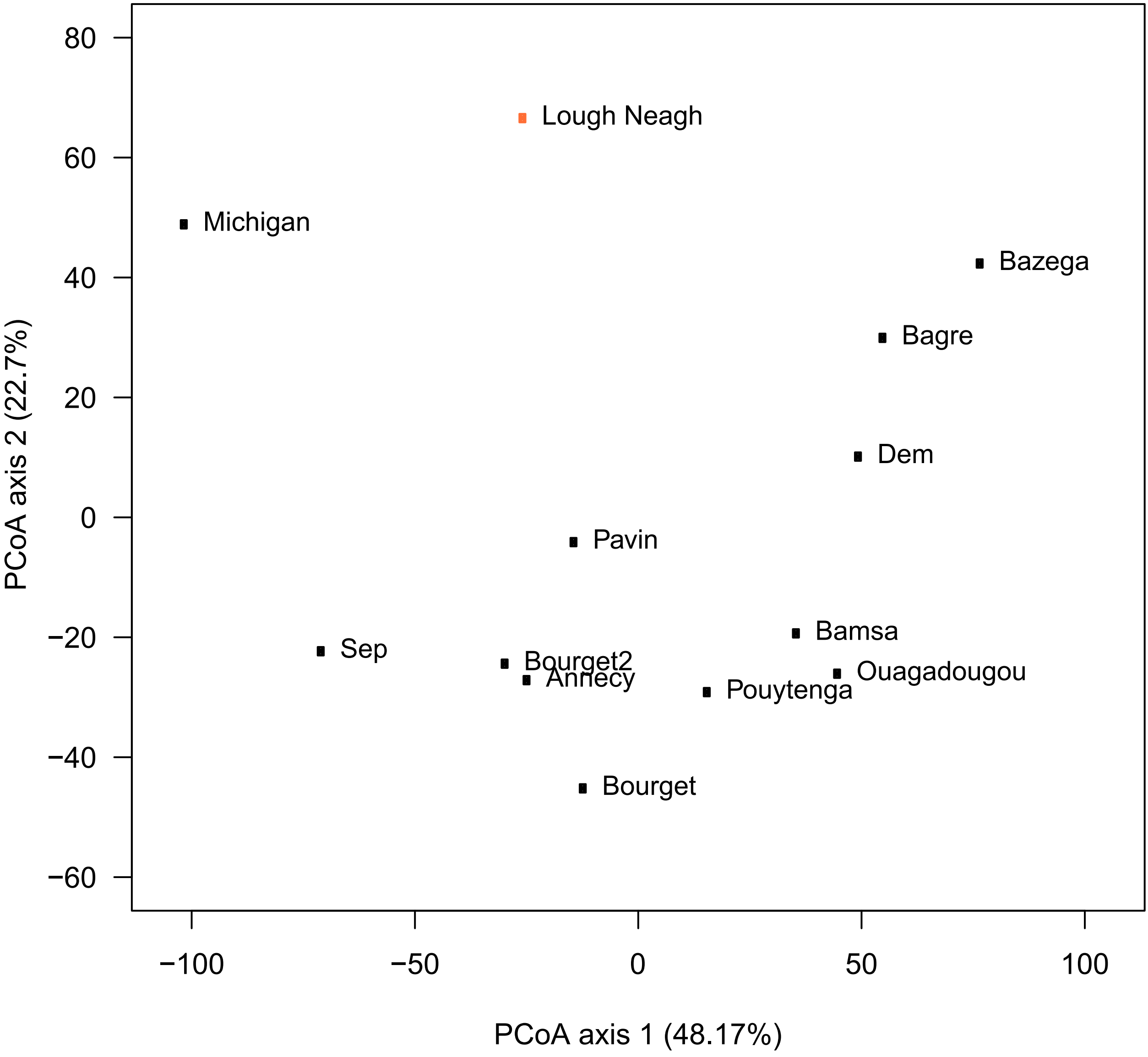

Lough Neagh
Michigan
Bazega
Bagre
Dem
Pavin
Bamsa
Sep
Bourget2
Annecy
Ouagadougou
Pouytenga
Bourget
80
60
40
20
0
−20
−40
−60
−100
−50
0
50
100
PCoA axis 2 (22.7%)
PCoA axis 1 (48.17%)
